# Supplementary material for: AWA and ASH Homologous Sensing Genes of Meloidogyne incognita Contribute to the Tomato Infection Process
Source: Pathogens. 2022 Nov 10;11(11):1322. doi: 10.3390/pathogens11111322 (PMC9693415; doi:10.3390/pathogens11111322)
Supplement: Supplementary file 1 [file pathogens-11-01322-s001.zip › pathogens-2010120-supplementary.pdf]

Table S1

Comparison of homologous genes between *Meloidogyne incognita* and *Caenorhabditis elegans*

Homology Type	*Meloidogyne incognita*	Gene Name
GPCRs	Minc3s01623g25167	*odr-10*
GPCRs	Minc3s00050g02698	*odr-7*
G protein	Minc3s01972g27520	*gpa-6*
G protein	Minc3s00003g00191	*gpa-11*
PLC	Minc3s00012g00794	*plc-1*
PLC	Minc3s05559g38432	*egl-8*
PLC	Minc3s00270g09074	*unc-13*
PLC	Minc3s00483g13114	*elo-1*
TRPV	Minc3s00057g02942	*osm-9*

Table S2

Comparison of protein homology between *Meloidogyne incognita* and *Caenorhabditis elegans*

Meloidogyne incognita	Gene Name	Protein Homology（%）
Minc3s01623g25167	*odr-10*	43.49
Minc3s00050g02698	*odr-7*	66.57
Minc3s01972g27520	*gpa-6*	41.76
Minc3s00003g00191	*gpa-11*	53.44
Minc3s00012g00794	*plc-1*	64.99
Minc3s05559g38432	*egl-8*	70.91
Minc3s00270g09074	*unc-13*	83.76
Minc3s00483g13114	*elo-1*	73.78
Minc3s00057g02942	*osm-9*	66.4

Table S3

Primer information of RNAi for *Meloidogyne incognita*

Meloidogyne incognita	Prime F	Prime R
Minc3s01623g25167	TAATACGACTCATATAGGGAGCTGGGCCTTCACAAAATGGA	TAATACGACTCATATAGGGAGAAACGTGGTGGTGCTGTTAA
Minc3s00050g02698	TAATACGACTCATATAGGGAGCAAACAACTCTAGTAGAACCAGC	TAATACGACTCATATAGGGAGGTCTTATCACGCACCGCTG
Minc3s01972g27520	TAATACGACTCATATAGGGAGGCGATTGTGGACTGATTCTGG	TAATACGACTCATATAGGGAGCATCGTTACGGCATCAAATACA
Minc3s00003g00191	TAATACGACTCATATAGGGAGCGGAGGGCACGTACTATAGA	TAATACGACTCATATAGGGAGTGTCCGCCAACATCTACCAT
Minc3s00012g00794	TAATACGACTCATATAGGGAGAAGAGCATGAACCCGATCCT	TAATACGACTCATATAGGGAGGAGAGGGCAATCTAGGGCTC
Minc3s05559g38432	TAATACGACTCATATAGGGAGTTTTATGCGACGTCCAGACC	TAATACGACTCATATAGGGAGTGCGCAGAAAGGTAAGCTTT
Minc3s00270g09074	TAATACGACTCATATAGGGAGTACAAAGAGCCGCCGAAAAG	TAATACGACTCATATAGGGAGCGAATTGCTCCGCTAACAACT
Minc3s00483g13114	TAATACGACTCATATAGGGAGTCTCGCCATTTTCTCAACCA	TAATACGACTCATATAGGGAGAGCAGACCCATTACTAGCCT
Minc3s00057g02942	TAATACGACTCATATAGGGAGAACGAAGCAAAATCCGCCTT	TAATACGACTCATATAGGGAGAAGCCTTCCATTTGTCAGCT

RT-qPCR primers for *Meloidogyne incognita*

Meloidogyne incognita	Prime F	Prime R
Minc3s01623g25167	CCATTGCTCTAAGTTGGGTTGT	AGCCTGAATGAGAAGAGTCCT
Minc3s00050g02698	CTCTGAACGTGTCCAAACCC	GTCTTATCACGCACCGCTG
Minc3s01972g27520	GCGATTGTGGACTGATTCTGG	GGTTCGTAATTGGGCTGTCC
Minc3s00003g00191	CGCATAACATGACCGAGGTG	GCCTTCTCCATACTGACACG
Minc3s00012g00794	TACCGGACGATGTACTCACC	GAAGAGGAGACACAGGCCAA
Minc3s05559g38432	TGCTGGTTGTCAAATGGCAG	GGTCTGGACGTCGCATAAAA
Minc3s00270g09074	CCTTCACAAGACCAACCTGT	TTGCCAGCGAAGATCATTGG
Minc3s00483g13114	ACGGATATTGGGTCTGGCTT	AATGCTGGCGTGATAGGGTA
Minc3s00057g02942	TGAATGTACAGCTACGCAG	AGAGGGTAAGCACTGCATGT

In situ hybridization primer for *Meloidogyne incognita*

Gene Name	Prime F	Prime R
gpa-6	GCGATTGTGGACTGATTCTGG	CATCGTTACGGCATCAAATACA
odr-10	CTGGGCCTTCACAAAATGGA	AAACGTGGTGGTGCTGTTAA
